# Diminished Value Discrimination in Obsessive-Compulsive Disorder: A Prospect Theory Model of Decision-Making Under Risk

**DOI:** 10.3389/fpsyt.2019.00469

**Published:** 2019-07-08

**Authors:** Sophie A. George, Jony Sheynin, Richard Gonzalez, Israel Liberzon, James L. Abelson

**Affiliations:** ^1^Veterans Affairs Ann Arbor Healthcare System, Ann Arbor, MI, United States; ^2^Department of Psychiatry, University of Michigan, Ann Arbor, MI, United States; ^3^Department of Social and Behavioral Sciences, Dixie State University, St George, UT, United States; ^4^Department of Psychology, University of Michigan, Ann Arbor, MI, United States

**Keywords:** obsessive-compulsive disorder, decision-making, prospect theory, risk aversion, positive outcome

## Abstract

**Introduction:** It has been hypothesized that people diagnosed with anxiety and obsessive-compulsive disorder (OCD) exhibit behavioral aberrations when faced with the potential for negative outcomes, but the specific cognitive aspects of decision-making that may be altered have not been systematically studied in clinical populations. Here, we studied decision-making in a clinical cohort using a task that allows for examination of the decision weights and values associated with different choice outcomes.

**Methods:** Patients diagnosed with OCD (*n* = 10), generalized anxiety disorder (*n* = 15), social anxiety disorder (*n* = 14), and healthy controls (*n* = 20) were given a decision-making task and choices were modeled using a cumulative prospect theory framework.

**Results:** We found OCD patients to have lower value discrimination than controls, as well as less optimal performance on the task, an effect that was mostly driven by trials with only positive outcomes.

**Discussion:** Our results shed light on the cognitive processes that drive altered decision-making under risk in OCD. Specifically, they demonstrate that OCD patients have diminished sensitivity to positive outcomes, which might be associated with risk aversion and altered learning of gain. These findings also extend prior reports, suggesting that altered cognitive processing during decision-making is linked to altered perception of value, but not probability, in these patients.

## Introduction

It has been hypothesized that people diagnosed with psychiatric disorders like obsessive-compulsive disorder (OCD), generalized anxiety disorder (GAD), and social anxiety disorder exhibit behavioral aberrations when faced with the potential for negative outcomes (1, 2). These behavioral tendencies may be shaped by specific cognitive-emotional processes, like greater attentional bias towards threatening stimuli (3), greater sensitivity to the possibility of loss (4, 5), and greater intolerance of uncertainty (6). These are also known to be among the factors that guide decision-making behavior when an individual makes a choice between several alternatives with different subjective values (7), implying that psychiatric disorders may alter normal decision-making processes in maladaptive ways.

**Figure 1:**
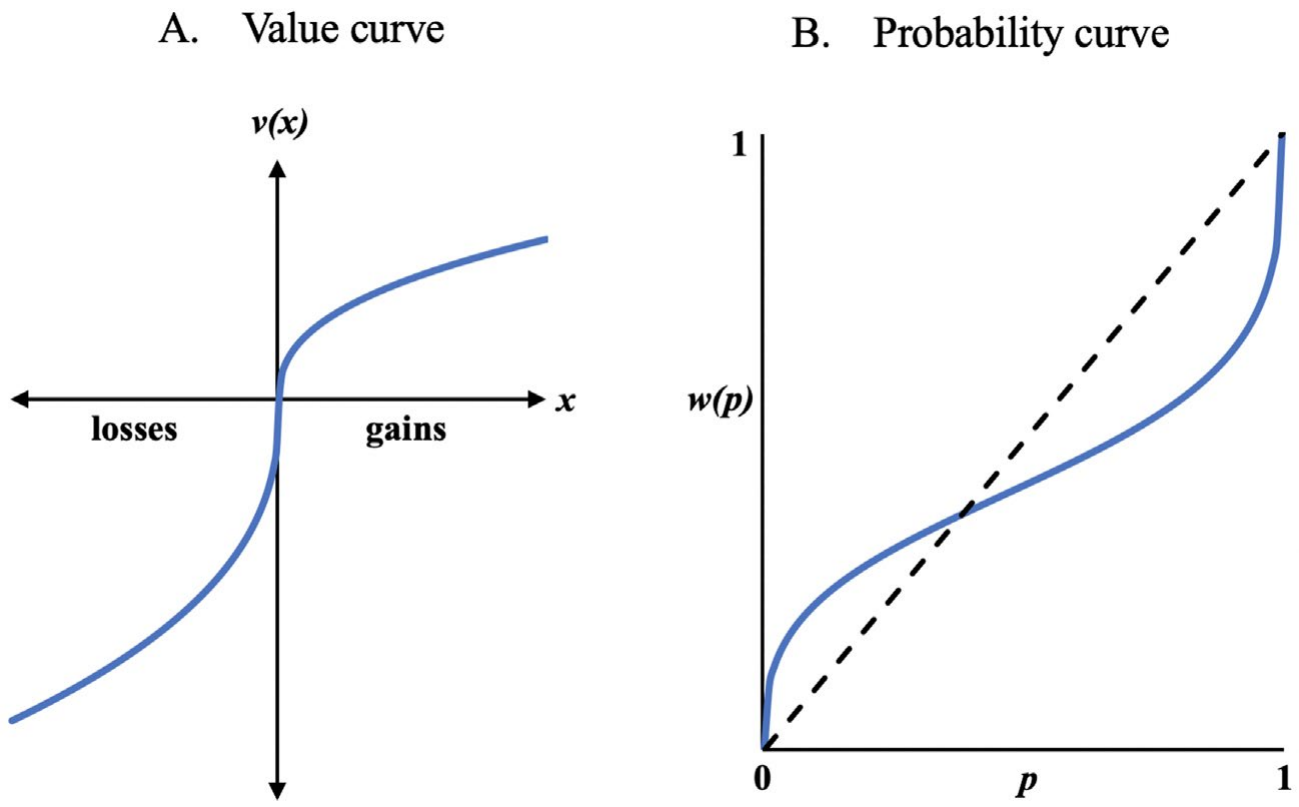
Classic prospect theory value **(A)** and probability **(B)** curves.

Recently, behavioral neuroeconomic tools have been touted as having high potential utility in assessing the decision-making characteristics of people with psychiatric disorders (1, 8, 9). Such an approach computes “optimal” or normative behavior on a variety of dimensions, thus allowing for precise quantification of deviation from these norms. The traditional view conceptualizes decision-making as a rational process involving simple comparisons of expected values or expected utilities. However, because human behavior routinely deviates from purely “rational” choice, cumulative prospect theory (10) offers empirically validated mathematical formulations of psychological effects in decision-making, such as loss aversion, and the circumstances when risk-seeking or risk-averse behaviors are likely to occur. Cumulative prospect theory posits that an individual’s choices can be described using an S-shaped value function (i.e., concave for gains and convex for losses; **Figure 1A**), which suggests a diminishing sensitivity to changing outcome values as they rise or fall (11). Subjective value increases rapidly for small increments in gains at low gain levels, but more slowly for similar value changes at high gain levels. Similarly, subjective value falls rapidly for small incremental losses at low loss levels, but more slowly for similar incremental losses at higher loss levels. This function also accounts for the loss aversion phenomenon, which suggests greater sensitivity to potential losses than to equivalent gains (12). The other component of cumulative prospect theory is an inverse S-shaped probability (weighting) function, which overweights low probabilities and underweights high probabilities (**Figure 1B**) (11). Here, the relationship between perceived probabilities and actual probabilities is fairly linear through the middle range of actual probabilities, but there is a tendency to subjectively overweight or underweight probabilities at the ends of this distribution curve.

Three recently published studies have applied a cumulative prospect theory approach to evaluate decision-making in clinical populations. Charpentier and colleagues (13) examined unmedicated patients with GAD and found that compared to healthy controls (HCs), patients had lower value discrimination (interpreted as “enhanced risk aversion” by the authors). Somewhat surprisingly, this study found no evidence of differences in loss aversion between the patient and control groups. Aranovich and colleagues (14) reported that OCD patients have lower sensitivity to outcome values (interpreted as “less risk aversion” by the authors), and an S-shaped probability function that contrasted with the classic inverse S-shape exhibited by controls. Further, Sip and colleagues (15) found that unmedicated OCD patients demonstrated greater loss aversion than medicated OCD patients and HCs. These findings provide the first evidence that people with anxiety disorders and OCD might exhibit differences in how they integrate value and probability information, but there are also important limitations to these studies. Charpentier et al. (13) and Sip et al. (15) tested only the value function of cumulative prospect theory, without manipulating the probability of the different outcomes. Conversely, Aranovich et al. (14) modeled the probability function, but did not manipulate the values of the different outcomes and did not include a gain–loss (“mixed”) condition to test loss aversion. In addition, none of the studies examined decisions within the loss domain (“loss-only” trials), and Sip et al. (15) did not include trials within the gain domain (“gain-only” trials). Inclusion of single domain trials in study design is required to examine the shape of the value function across all possible outcomes.

Some additional studies have examined risky decision-making in clinical populations using behavioral tasks such as the Iowa Gambling Task or Balloon Analogue Risk Task [e.g., Refs. (4, 16–21)], and some studies applied a prospect theory inspired perspective to their findings [e.g., Ref. (22), which focused primarily on learning]. While these studies can reveal patterns of risky decision-making, these reports do not provide information about the specific factors that drive such patterns (e.g., altered perception of value, altered perception of probability, or both) because they did not manipulate the outcomes and probability in a manner that permits decoupling these factors.

The present study, therefore, aimed to fill these gaps by employing a comprehensive cumulative prospect theory experimental design, which varies both outcomes and probabilities, using gain- and loss-only trials, as well as mixed trials. We used the Michigan Decision-Making (MDM) task previously developed in our laboratory (23), which involves a series of stochastic decisions, in which participants choose to play one of two possible gambles. The number of points that can be won or lost and the probability of each outcome are displayed on-screen. This allows for the simultaneous estimation of parameters describing sensitivity to change in outcome values (“value discrimination”), the loss aversion parameter, and the curvature of the probability weighting function. We hypothesized that alterations in decision-making parameters would be found in patients, consistent with the greater risk and loss aversion often reported. The purpose of such a comprehensive investigation was to determine the precise nature of the decision-making alterations, which might drive the effects seen in other studies that were not able to calculate all decision-making parameters suggested by prospect theory.

## Methods

### Participants, Diagnoses, and Clinical Measures

This was a naturalistic study of treatment-seeking patients with anxiety presenting to the Anxiety Disorders Clinic at the University of Michigan. All patients presenting for an initial clinic evaluation were eligible for the study. Thirty-nine participants with a current principal *Diagnostic and Statistical Manual of Mental Disorders*, 4th edition (DSM-IV) diagnosis (24) of GAD (38.5%), social anxiety disorder (35.9%), or OCD (25.6%) were enrolled (**Table 1**). The majority of the patients (69.2%) also had one or more comorbid diagnoses, primarily of anxiety disorders [panic, GAD, OCD, social anxiety, posttraumatic stress disorder, or anxiety not otherwise specified (NOS)] and/or mood disorders (major depressive disorder, dysthymia, or depression NOS). All participants’ diagnoses were established using clinical consensus in a multidisciplinary team meeting of experienced clinicians (with >50 years of cumulative experience in anxiety diagnoses and >20 years of cumulative experience using structured interviews to establish research diagnoses). To verify concordance between clinical consensus diagnoses and diagnoses based on structured clinical interviews (SCID) (25), this team periodically performs SCIDs on subsets of patients enrolled in all studies, and consistently finds kappas scores > .90. Accordingly, 10 of the randomly assigned patients enrolled in this particular study received both clinical consensus diagnoses and were interviewed with the SCID; in all cases, the clinical consensus primary diagnosis was confirmed. Agreement about the presence of comorbidity was also high, but the specific secondary and tertiary diagnoses from the clinic were less reliably confirmed by SCID and were not utilized in analyses. Exclusion criteria included: 1) suicide risk; 2) gross cognitive impairment or physical disability that might render participation or completion of study tasks difficult; 3) psychosis; and 4) newly (past 4 weeks) prescribed or changed psychiatric medications. Many of the patients were on medications: *n* = 19 on eight different selective serotonin reuptake inhibitors (SSRIs), *n* = 7 on three different benzodiazepine in varying patterns (mostly short term and as needed), and *n* = 3 on three different stimulants.

**Table 1:** Participant demographic and clinical characteristics.

Primary diagnosis	N	Mean age years (SD)	Gender (% female)	PHQ* mean score (SD)	WSAS* mean score (SD)	GAD* mean score (SD)	BDI mean score (SD)
Generalized anxiety disorder	15	34.5 (15.2)	73.3	10.2 (6.6)	19.1 (11)	14.5 (6)	22.1 (13.5)
Obsessive-compulsive disorder	10	27.5 (9.7)	40.0	11.6 (8.7)	22.1 (9.4)	11.5 (6.6)	22.8 (13.9)
Social anxiety disorder	14	30.8 (11.6)	28.6	10.1 (6.2)	20.4 (8.1)	11.8 (5.1)	16.4 (8.5)
Healthy controls	20	25.6 (6.9)	55.0	1.3 (2.2)	.1 (.7)	.9 (1.4)	2.7 (4.9)

*There was one missing Patient Health Questionnaire-9 (PHQ-9) score, one missing Work and Social Adjustment Scale (WSAS) score, and two missing generalized anxiety disorder (GAD-7) scores. No Beck Depression Inventory (BDI) scores were missing.

Twenty HC participants (55% male, mean age 25.6 years, *SD* = 6.89) were also recruited from the community. HCs were screened with SCID-NP and excluded if there was any history of major psychiatric disorder or current substance abuse. All patients evaluated in our anxiety clinic completed a standard battery of self-report clinical severity measures. These include three symptom measures—the Patient Health Questionnaire-9 (PHQ-9) (26), the Generalized Anxiety Disorder-7 (GAD-7) (27), and the Beck Depression Index (BDI) (28); and one functional measure—the Work and Social Adjustment Scale (WSAS) (29). All HCs were also asked to complete these measures at time of enrollment. This study was carried out in accordance with the recommendations of the American Psychological Association for the ethical treatment of research participants. The protocol was approved by the Institutional Review Board of the University of Michigan Medical School. All participants gave written informed consent in accordance with the Declaration of Helsinki.

### Experimental Task

**Figure 2:**
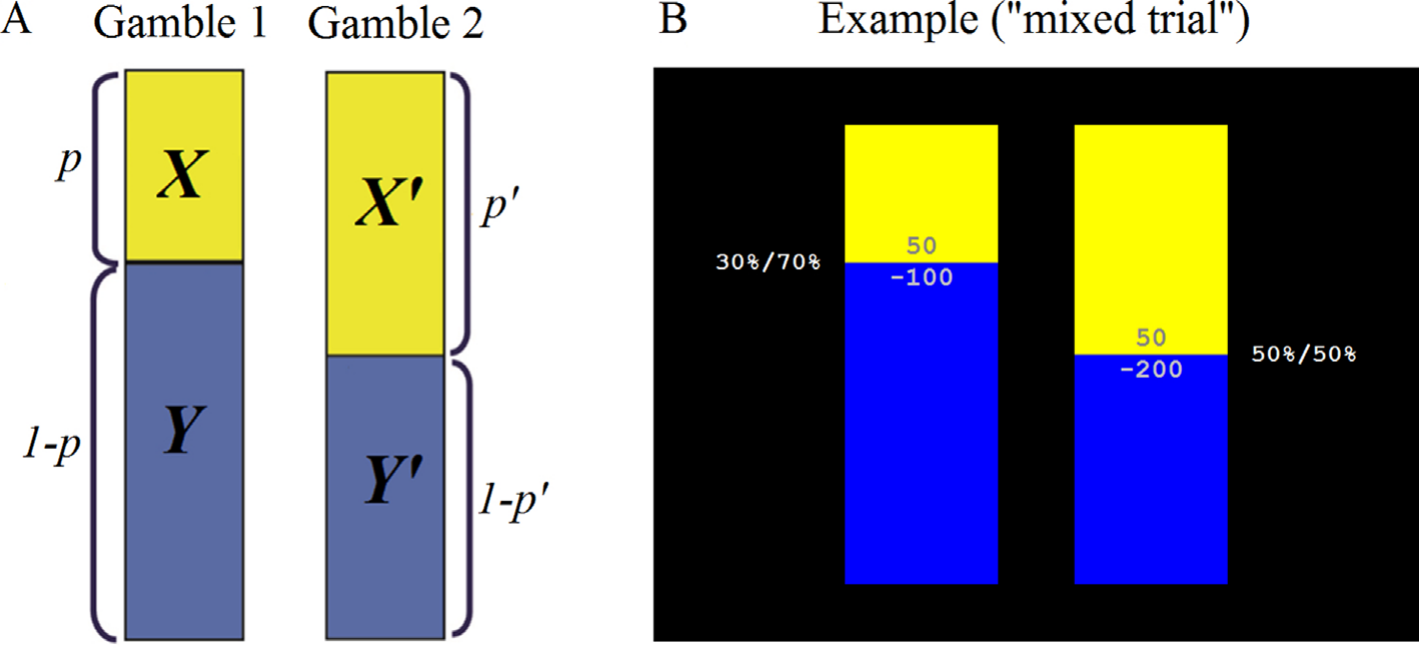
Michigan Decision-Making (MDM) task. **(A)** Schematic representation: The task consisted of 126 trials, composed of two gambles each. Each gamble had two possible outcomes with values *x* and *y* and probabilities *p* and 1-*p*, respectively. **(B)** Screenshot of an example “mixed trial.”

The MDM task consisted of 126 trials, composed of two gambles each. Each gamble had two possible outcomes with values *x* and *y* and probabilities *p* and *1-p*, respectively (**Figure 2**). The two gambles in a trial are denoted as (*x, y, p*) and (*x’, y’, p’*). The values ranged from −200 to 200, and five probability levels were used (.05, .30, .50, .70, .95). The gambles were presented graphically, with the outcome values printed within colored bars (yellow, blue), and the length of the colored bars corresponding to the probabilities, which were also presented in numerical form beside the bars. Trials were classified as Gain-Only (where *x, y, x’, y’* ≥ 0), Loss-Only (where *x, y, x’, y’* ≤ 0), and Mixed-Outcome (where in each gamble outcomes have opposite signs) and were randomly intermixed with each trial type appearing 42 times. On each trial, the participant had up to 5 s to decide between outcomes. A fixation cross was presented during the intertrial interval (4–10 s long, mean 5.5 s). At the end of the task, the total number of points accumulated was displayed on the screen and participants were paid the appropriate amount.

While there was no practice block, the task started with the following on-screen instructions to ensure the participant’s understanding of the task: “Welcome to the gambling task. In this task, you are to make a choice between two gambles at a time. That is, you choose to play one gamble and not play the other one. Each gamble is associated with two possible outcomes (known as points). If you ‘play a gamble’, you will have a chance (for example, 5%, 30%, 50%, 70%, 95%) to get one of the two possible outcomes, or get the other outcome otherwise. Graphically, each gamble is presented as a combination of BLUE and YELLOW bars. Each color bar contains one possible outcome. The length of a color bar represents the chance of getting the outcome in the bar (longer bar means more likely to get the outcome inside). You will also see the chances in percentages written on the side. This is what a gamble looks like [graphical example with explanation]. Each time you will see two gambles [graphical example]. To choose a gamble to play: Press ‘2’ key to play the gamble on the left-hand side. Press ‘3’ key to play the gamble on the right-hand side. Your goal is to accumulate as many points as possible. While every gamble you play will be counted by the computer accordingly, no immediate feedback will be displayed. In the end, the total points will be displayed. Please pay attention to ALL the outcomes and bar lengths before you choose a gamble to play. You have only 5 seconds for each choice, please answer as soon as possible. If your response is too slow, you will lose 5 points. This is the end of the instructions. Any questions?” The participant was then instructed to press a key to proceed to the first trial.

### Experimental Procedures

Testing took place between 0900 and 1800 h. On arrival in the laboratory, participants gave informed consent and completed a battery of questionnaires, followed by three behavioral tasks, all of which were administered on a computer. The three tasks were the MDM, Probabilistic Selection Task (PST) (30), and Approach-Avoidance Conflict (AAC) task (31). Task order was counterbalanced and only data generated from the MDM task are reported here. The MDM task was programmed and run on E-Prime 2.0 SP1 (Psychology Software Tools Inc., Sharpsburg, PA). Participants were told that while no feedback would be given during the task about performance, the outcomes of all trials would be summed and converted to a monetary reward on a predetermined scale, resulting in a “bonus” payout of between $0 and $20 upon completion. This bonus amount was in addition to the $15 they were paid for participating in the experiment. Following completion of the experimental procedures, participants were debriefed.

### Cumulative Prospect Theory Model

Choices on the MDM task were modeled within the cumulative prospect theory framework (10). The cumulative prospect theory model includes two main components: the value function and the probability weighting function. The standard form of the value function *v* is a simple power relation that also considers the sign of the outcome:

(1) $$\begin{array}{cc} v(x)=x^{\alpha } & \text{if }x\geq 0 \end{array}$$

$$\begin{array}{cc} -\lambda {\left(-x\right)}^{\alpha } & \text{if }x<0 \end{array}$$

Following the framework outlined in this seminal cumulative prospect theory paper, we estimated equal curvature parameters in the gain and loss domains (α; “value discrimination”) (10). The loss aversion coefficient (λ) is a differential weighting parameter, which comes into play in choices between Mixed-Outcome gambles because if all the outcomes are negative, it will cancel out in binary choice.

While several forms of the probability weighting function *w* have been reported, two-parameter functions were shown to outperform common single parameter functions (32). These functions typically cannot be distinguished with small datasets, and the form used here is:

(2) $$w(p)=\delta p^{\gamma }/(\delta p^{\gamma }+{\left(1–p\right)}^{\gamma })$$

The parameter γ controls the curvature and the parameter δ controls the elevation. For the case of choices between Gain-Only gambles, the function ƒ representing cumulative prospect theory becomes:

(3) f(X)=w(p)v(x)+(1−w(p))v(y)

where *X* contains three dimensions (one for each outcome and one for the probability *p* of obtaining the more extreme outcome with *x* and, implicitly, probability 1–* p* for obtaining the other outcome *y*). The form of ƒ is similar for Losses-Only gambles, but for Mixed-Outcome gambles it changes to:

(4) f(X)=w(p)v(x)+w(1−p)v(y)

The binary choice between a pair of gambles *A* and *B* is a function of the difference ƒ(A)–ƒ(B). Here, we use the following logistic function to map the difference between two utilities to the [0, 1] scale:

(5) P(choosing A over B)=1/(1+exp(k(f(B)–f(A)))

We used the special case choice function with *k* fixed to 1, so as not to include additional non-cumulative prospect theory parameters in the estimation. Our goal was to use cumulative prospect theory to guide comparisons across the four groups of participants, and we wanted to use the restrictive form of the model consistent with the Tversky and Kahneman (10) paper. This choice function can be fit with standard regression techniques. In the present paper, we implemented the estimation in the statistical package R, within RStudio version 0.99.441 development environment (RStudio Inc., Boston, MA), and obtained the estimated parameters using a nonlinear mixed effects (nlme) model fit to estimate heterogeneity across participants (33). We estimated both fixed and random effects for each parameter in each participant. We used dummy parameters to estimate additional fixed effects for each participant group. Specific values that were used to optimize model fit are detailed in the **Supplementary Material**.

After obtaining the estimated cumulative prospect theory parameters from our model, we followed up by performing *post hoc* analyses on the behavioral data to assess performance relative to a rational model. Specifically, we examined the simplest decision-making strategy, according to which individuals choose gambles with highest expected values (EV):

(6) EV=(p)(x)+(1−p)(y)

We calculated the difference between the EVs of the chosen gamble and the non-chosen gamble (ΔEV). Higher ΔEV represents more optimal behavior (or less suboptimal), which should result in a better outcome. We expected our behavioral data to be in line with the findings obtained from the cumulative prospect theory model.

### Statistical Analysis

Statistical analyses were conducted using IBM SPSS Statistics version 24. Independent samples *t*-tests were performed to test differences in estimated parameters between the three patient groups and HC, for each one of the four cumulative prospect theory parameters (total of 12 *t*-tests). In *post hoc* behavioral analyses, we used mixed analyses of variance (ANOVA) with within-subject factor of trial (14 “Gain-Only” or “Loss-Only” trials where both gambles had identical probabilities) and between-subject factor of group (each patient group vs. HCs). Alpha was set to 0.05 (two-tailed) with Bonferroni correction used to protect against inflated risk of family-wise error. Effects and interactions with *p* > .100 were not reported.

## Results

There were no significant differences between HCs and each of the three patient subgroups in age or gender (**Table 1**; Bonferroni corrected α-value 0.05/6 = .008; all *p’s* ≥ .048). As expected, there were differences on the self-report clinical measures (PHQ-9, GAD-7, BDI, and WSAS). All patient subgroups had elevated scores on these four measures (see **Table 1**) relative to HCs (corrected α-value 0.05/12 = .004; all *p’s* < .004, except difference between HC and OCD on PHQ-9, which did not reach corrected significance; *p* = .007). There were no significant differences between the three patient subgroups on any of these measures, as well as on the ratio of patients with comorbid diagnoses, and the ratio of patients using each one of the medication types (all *p’s* > .10).

**Figure 3:**
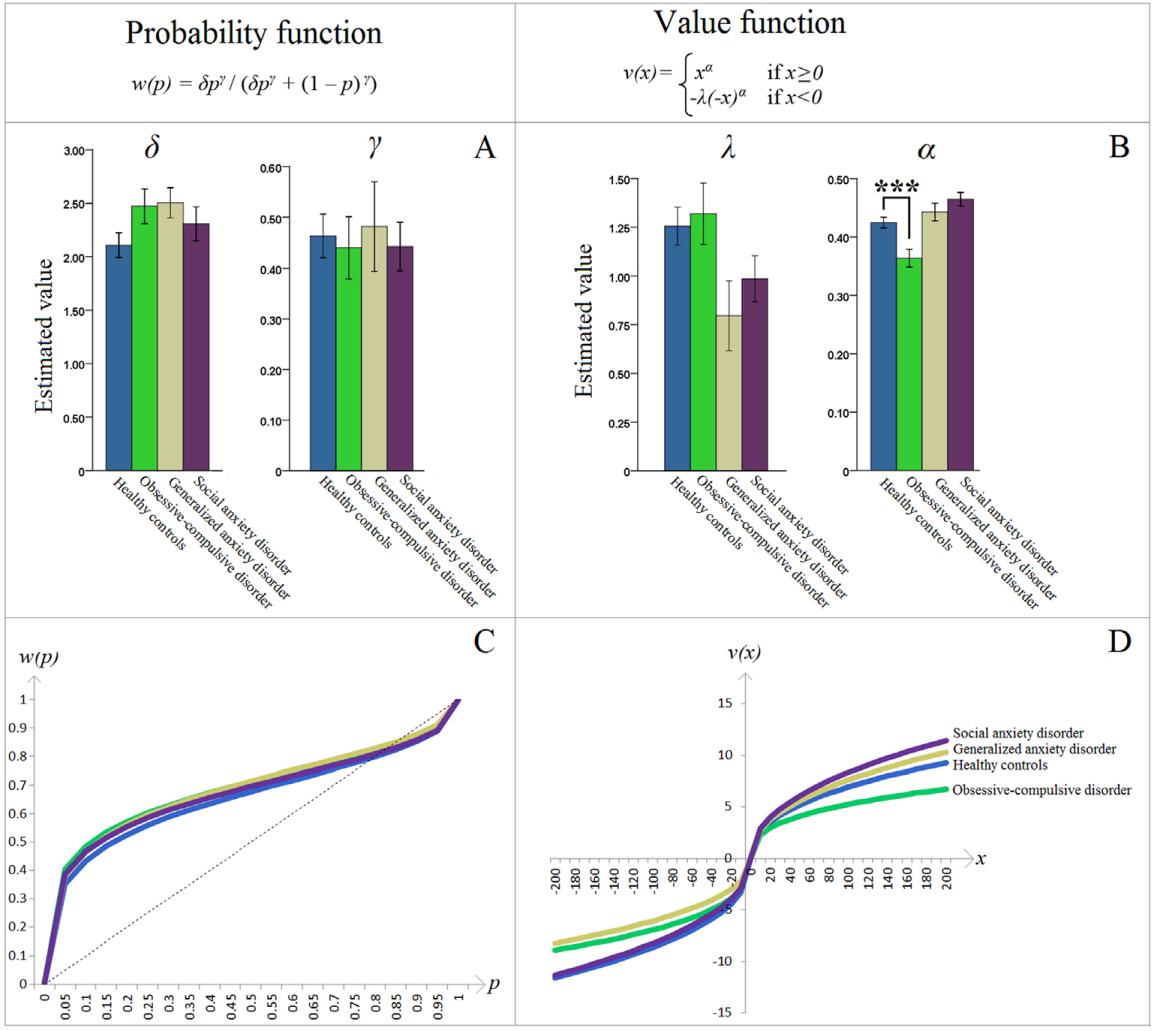
**(A)** Estimated parameters of the cumulative prospect theory (CPT) probability weighting function: δ controls the elevation and γ controls the curvature. **(B)** Estimated parameters of the CPT value function: λ is the loss aversion coefficient and α controls the curvature (“value discrimination”). Asterisks (***) represent significant difference between groups (*p* = .001). Error bars indicate ±1 standard error of the mean. The obsessive-compulsive disorder–healthy control (OCD-HC) difference in Panel B is further dissected in **Figure 4**. **(C**, **D)** The resulting CPT probability *w*(*p*) and value *v*(*x*) functions, respectively, based on the group means depicted in **(A**, **B)**. Specifically, the *y*-axes represent the decision weights and values, respectively, while the *x*-axes show actual increments in probabilities and values. For additional explanation, see the second paragraph in the Introduction section.

There was a significant difference between OCD patients and HCs on the estimated cumulative prospect theory parameter α (“value discrimination”) [*t*(28) = 3.623, *p* = .001, *d* = 1.364]; OCD patients had lower α than HCs (**Figure 3B**). No other significant differences between HC and patient groups on any of the other parameters were found (corrected α = .050/12 = .004; all *p*’s > .008) (**Figures 3A, B**). Of note, analyses of fixed effects tests from the nonlinear mixed effects estimation procedure similarly showed a significant effect of lower value discrimination in the OCD group [*t*(7360) = −1.969, *p* = .049]. These fixed-effects analyses also showed lower loss aversion in the GAD group [*t*(7360) = −2.102, *p* = .036], but since it was not supported by the corresponding Bonferroni corrected *t*-test (*p* > .008), it was deemed not significant.

Interestingly, only the HC group had λ values different (greater) than 1 [one-sample *t*-test, mean (*SD*) = 1.255 (.437), 95% *CI* (1.050, 1.459), *t*(19) = 2.604, *p* = .017, *d* = .582]. In the OCD group, λ was numerically greater than 1, but this did not reach statistical significance [mean (*SD*) = 1.319 (.499), 95% *CI* (.962, 1.675), *t*(9) = 2.021, *p* = .074, *d* = .639].

Probability and value functions were plotted, based on the fixed effect (i.e., mean) of the estimated cumulative prospect theory parameters within each participant group. Groups were visually indistinguishable on the probability function (**Figure 3C**). However, the OCD group had a distinctly different curve on the value function (**Figure 3D**). Specifically, this group had lower absolute cumulative prospect theory values, relative to HCs, on both the loss and gain domains. This visually confirms, in the standard cumulative prospect theory curves, the significant statistical difference described above.

**Figure 4:**
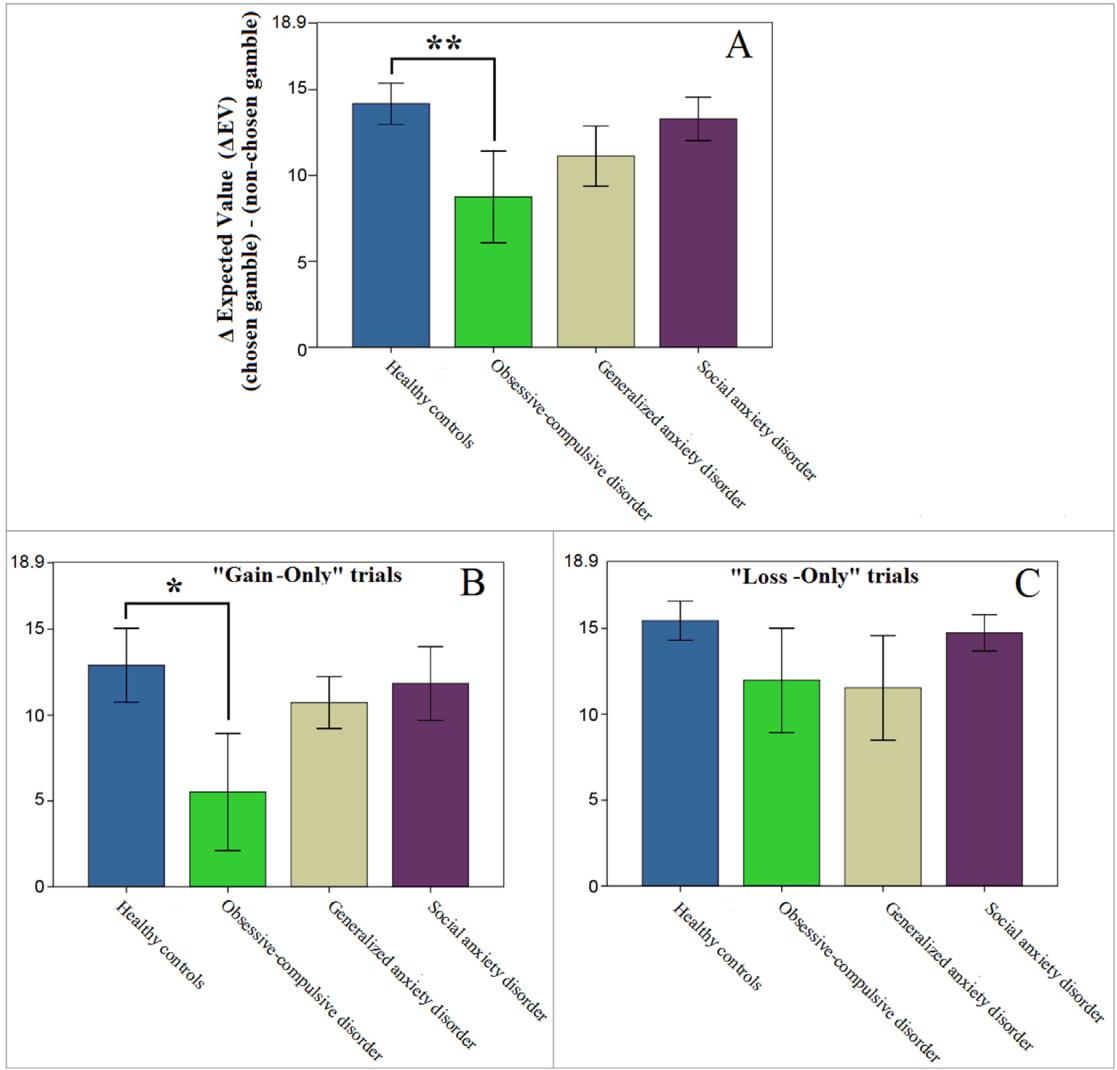
Performance on “Gain-Only” and “Loss-Only” trials where both gambles had identical probabilities (14 out of 126 trials). Higher values represent more optimal performance with potentially better outcomes. Note that the maximum value on the *y*-axes (18.9) represents the optimal difference in expected value (ΔEV), if the gamble with the higher EV was always chosen. **(A)** Patients diagnosed with obsessive-compulsive disorder (OCD) showed less optimal performance than controls (**p = .041). **(B**, **C)** This result appeared to be mostly driven by differences on “Gain-Only” trials (*p = .067, whereas *p* = .306 for the OCD vs HC comparison on “Loss-Only” trials). Error bars indicate ±1 standard error of the mean.

To confirm the results from our model estimations we followed up with exploratory (uncorrected) *post hoc* analyses of the behavioral data. Since group differences were found only within the value function domain, we controlled for probability weighting by selecting only the trials with identical probabilities (17 out of 126). Further, to focus on value discrimination, we excluded three Mixed-Outcome trials, where behavior could be affected by loss aversion. If indeed value discrimination in OCD patients is lower than in HC, their sensitivity for high gains and/or losses should be diminished, which would result in less optimal performance on these trials (as assessed by the difference in the expected values of the chosen and the non-chosen gambles; Equation 6). This is exactly what we found; only the OCD patients showed suboptimal performance on these trials [mixed ANOVA, main effect of group; *F*(1,28) = 4.608, *p* = .041, *d* = .766; **Figure 4A**], which was driven primarily by their decisions on the “Gain-Only” trials [*F*(1,28) = 3.640, *p* = .067, *d* = .724; **Figures 4B, C**].

While there were no significant group differences in age or gender between HC and any of the patient groups, we reran the comparisons of the estimated parameters while controlling for age and gender and results were unchanged, i.e., the only difference that reached significance was between HC and OCD groups on the α parameter (*p* = .001). To test for potential medication effects, we also compared estimated parameters between patients on SSRIs and those not, as well as patients on benzodiazepines and those who were not. These differences did not reach significance (all *p’s* > .400). Lastly, we examined correlations between our estimated model parameters and clinical measures across all patients (*n* = 39; corrected α-value 0.05/16 = .003). WSAS score was correlated with the probability parameter γ (*r* = −.468, *p* = .003), as well as with the loss aversion coefficient λ (*r* = .481, *p* = .002). The probability parameter δ, and the value discrimination parameter α, were not correlated with any measure (all *p’s* > .010).

## Discussion

The present study used a cumulative prospect theory framework to characterize decision-making in a naturalistic sample of patients diagnosed with OCD, GAD, and social anxiety disorder. Our aim was to confirm and extend earlier reports demonstrating decision-making abnormalities in patients with psychiatric disorders by using a comprehensive task that allows for the simultaneous estimation of parameters describing value discrimination, loss aversion, and the weighting of probabilities. Application of a comprehensive task like this to patient populations has not, to our knowledge, been previously reported. We found that OCD patients show lower value discrimination relative to HCs. This suggests that people with OCD are less sensitive to increasing values of potential gains or losses. Specifically, in our experimental paradigm, lower value discrimination results in participants being less likely to choose to pursue greater gains or to avoid greater losses when outcomes involve higher values. This could, in turn, lead to greater risk aversion in the context of potential gains, but also greater risk acceptance in the context of potential losses. Subsequent behavioral analyses were in line with these model simulations and indicated that only the OCD group showed suboptimal task performance, a difference that was driven by performance primarily on the gain-only trials. This suggests that lower value discrimination in the gain domain may contribute to altered decision-making in OCD patients. Specifically, as gains mount, OCD patients do not attach as much value to each additional increment of gain as do HCs, and are perhaps thus less motivated to pursue the potentially available monetary reward. Interestingly, no other significant differences were found, suggesting that differences in decision-making on this task are specific to the value discrimination parameter and to individuals diagnosed with OCD, at least within the statistical power of the present study.

The finding of significantly lower value discrimination (termed α here) in OCD patients compared to HCs is in agreement with a recent report from Aranovich and colleagues (14) who identified less “outcome sensitivity” in these patients. It is also partially consistent with Sip et al. (15) who reported a flatter increase in the likelihood of accepting a gamble with increasing gains in unmedicated OCD patients, though their study focused exclusively on loss aversion and was not optimized to model value discrimination. These findings shed light on how previously identified dysfunctional reward circuitry in OCD patients, in particular in the anticipation of reward receipt (34), might manifest behaviorally and drive altered decision-making. Further evidence that altered value discrimination results in suboptimal decision-making comes from our *post hoc* analyses of the behavioral decision-making data. These analyses examined performance on “gain-only” and “loss-only” trials in which both gambles had the same probability, to isolate the effects of value while holding probability constant, as well as excluding the possibility that loss aversion might influence decisions. This approach demonstrated that decision-making in OCD patients resulted in suboptimal performance, as evidenced by a larger deviation from choices with the maximum expected value. Further, while the α value determines the curvature of the value function in both gain and loss domains, a visual inspection of the value function in **Figure 3D** indicates that the largest deviation between OCD and other groups occurred in the gain portion of the curve. Moreover, our behavioral findings indicate a clearer between-group difference in the “gain-only” trials (as depicted in **Figure 4B**). These data suggest that OCD patients had lower value discrimination, primarily in the processing of potential gains. This is consistent with the altered reward learning reported in OCD patients who performed the Iowa Gambling Task (16, 35), and is one possible reason why some studies that did not include gain-only trials failed to find similar group differences [e.g., Ref. (15)].

Clinically, lower value discrimination might relate to anhedonia in OCD patients, which has recently been demonstrated to be associated with OCD symptom severity and is suggested to be independent of comorbid depression (36). Lower value discrimination may also contribute to the excessive avoidance behavior demonstrated in OCD patients (37), since undervaluation of positive outcomes might result in predominance of avoidance behavior in a dynamic approach–avoidance system in which approaching gains must be balanced with avoiding potential losses (38).

In addition to capturing value discrimination, the value function also accounted for the loss aversion phenomenon, measured by the coefficient λ. The λ value shown by our HCs was statistically greater than 1, which is suggestive of “normal” loss aversion in this group and consistent with the initial proposition by Kahneman and Tversky (39). Interestingly, we found no evidence of abnormal loss aversion in the OCD patients. It is somewhat surprising given the disorder’s clinical presentation in which obsessions with fears of negative events drive compulsive responses aimed at preventing these events. To our knowledge, only one other study so far has specifically examined loss aversion in OCD, and there, unmedicated (but not medicated) OCD patients were found to have greater loss aversion than HCs (15). In our study, about half of the OCD patients were receiving stable doses (≥4 weeks) of antidepressant treatment. This might explain the lack of a significant loss aversion effect in our OCD patients and suggest that reduced loss aversion could be a mechanism of drug efficacy in OCD. However, if the “normalized” loss aversion seen in our study was due to medication, it would suggest that loss aversion and value discrimination were separable phenomena in OCD and that even with reduced loss aversion patients could still be symptomatic. This raises the question of whether separate but simultaneous pharmacological targeting on both loss aversion and value discrimination might produce better clinical outcomes. While we did not find evidence for medication effect, the relationship between loss aversion and value discrimination, and their shared and distinct pharmacological sensitivities, warrant examination in future studies.

Another decision-making concept is risk aversion, which represents the preference for a sure outcome over an uncertain prospect with equal or greater expected value (40). Several studies attempted to test risk aversion in OCD patients, but methods and findings have not been consistent. For instance, Sip et al. (15) conducted *post hoc* analyses to test whether differences in risk aversion contributed to the greater loss aversion found in unmedicated OCD patients. They found that these patients’ increased likelihood of rejecting a gamble as its loss value increased could not be explained solely by greater risk aversion. Similarly, while Pushkarskaya et al. (19) showed that OCD patients had higher avoidance of uncertain options, they did not appear to differ in their attitude toward risk. Lastly, Aranovich et al. (14) found less “outcome sensitivity” in OCD patients, which was interpreted as less risk aversion in these patients. Overall, these findings argue against a view of OCD as a risk aversion disorder (4). However, it is also important to note that risk aversion attitudes depend on sensitivities to both values and probabilities (see the fourfold pattern of risk attitudes) (10) and can be most accurately calculated when both probability and value functions are measured. Ours is the first study to simultaneously examine both functions in OCD, and the lack of differences on the probability function directly confirms the suggestions of normal risk perception in OCD, as suggested by other studies [e.g., Ref. (19)]. However, the lower value discrimination that we found suggests that risk avoidance in OCD might be driven, in an interesting but complicated way, by patients’ abnormal value perception (41), seen in our data primarily as an inappropriate undervaluing of the potential gains that might make risk taking worthwhile. Clearly, considerable additional work is needed to further test and explore this hypothesis; but, if true, it may add an important additional dimension for study in ongoing efforts to unravel the psychobiology of OCD.

We also examined decision-making in two additional patient groups—GAD and social anxiety disorder—and failed to find evidence of decision-making abnormalities in these anxiety disorders. There is a broad literature that suggests that people with anxiety have altered processes that affect decision-making (1, 2), such as a bias toward threatening stimuli (42), risk aversion (5, 17, 18, 20, 21, 43), and possibly loss aversion (1). However, there is also contrasting evidence. For instance, levels of loss aversion were not found to be altered in GAD patients or highly anxious adolescents, relative to HCs [Refs. (13, 44), respectively]. Further, paradigms that test risky decisions often use purely behavioral variables, such as ratio of risky gambles (4, 17–19) or number of risky responses (as in the Balloon Analogue Risk Task) (20, 21). While identifying risky behavior patterns is important, such reports often do not provide information about the specific cognitive factors that drive such patterns (e.g., altered perception of value, altered perception of probability, or both). Our work focused on mechanisms, rather than just behavior, and suggests that the decision-making mechanisms measured through a full cumulative prospect theory analysis are not abnormal in GAD and social anxiety disorder. However, our sample sizes were small and replication is needed in larger samples.

The strength of our study is that we used a paradigm designed to test the full range of parameters thought necessary to understand human decision-making behavior, based on cumulative prospect theory (10). Other clinical studies have commonly used tasks that lack gain and loss (i.e., “mixed”) trials, which are needed to examine loss aversion directly [e.g., Ref. (14)], they have not manipulated outcome probabilities to examine the probability function [e.g., Refs. (13, 15, 18)], or they have not included both “gain-only” and “loss-only” trials to test the full shape of the value function [e.g., Refs. (13, 15)]. Without examination of both value and probability functions, complete conclusions about risk attitudes cannot be drawn. By including all of the cumulative prospect theory-based elements, we were able to more thoroughly characterize the factors contributing to decision-making abnormalities.

The present study has a number of potential limitations. First, the sample was composed of “volunteers” from a treatment-seeking population and may not be representative of the disorders in general. However, participants were “real” patients rather than advertisement recruited subjects. Second, we did not have clinician administered symptom measures like the Yale–Brown Obsessive-Compulsive Scale or Hamilton Anxiety Rating Scale. Instead, we relied on self-administered symptom measures. Additionally, as is typical in the clinical setting, the majority of our patients (54%) were taking psychotropic medications (49% on SSRIs; 18% on benzodiazepines; and 8% on stimulants). While these proportions are in line with those reported in other similar studies [e.g., Ref. (14)], given earlier work indicating a medication related effect on loss aversion in OCD patients (15), medication status may prove to be an important factor. Although we did not find evidence for a medication effect, future studies specifically designed to identify potential interactions between psychotropic medications and decision-making are important. Further, a substantial proportion of subjects had comorbid mood and anxiety disorders. Comorbidity is a chronic challenge in studies like this. Including only “pure” cases is not only difficult but it may actually reduce generalizability because co-morbidity is so common clinically. Nevertheless, conclusions about specific diagnoses might be confounded. We also found fairly limited connections between clinical measures and cumulative prospect theory parameters (WSAS was related to loss aversion and the probability parameter γ). Value discrimination (α), which was the focus of our discussion, was not correlated with clinical measures even though OCD and HC participants differed on this parameter. Finally, while the small sample size in our study limited our ability to further dissect effects of potential interest such as medication, comorbid diagnoses, and a more detailed examination of clinical dimensions and decision-making, our report of significant effects in a small group of OCD patients suggests a potentially robust finding in value discrimination. The experimental design used many trial repetitions to generate power, resulting in parameter estimates with relatively narrow confidence intervals. This suggests that the reported results were consistent across participants and are likely to be replicated.

To our knowledge this is the first study to investigate decision-making abnormalities in a “decision-making under risk” framework in which the full potential of cumulative prospect theory to delineate the factors that contribute to decision-making has been deployed. Our study clarifies some gaps in understanding of the factors that drive decision-making in patient populations, and helps us more accurately interpret previously reported findings. The results suggest that altered decision-making in OCD is associated with alterations in the processing of outcome values and may be driven by abnormal learning of positive outcomes. This, in turn, might lead to the increased risk aversion and anhedonia often reported in these patients. This work also highlights the need for standardization of how the cumulative prospect theory framework and decision-making terminology are applied to psychological concepts to aid in the comparison of findings, and design of future studies that can build on earlier work.

## Data Availability

The datasets generated for this study are available on request to the corresponding author.

## Ethics Statement

This study was carried out in accordance with the recommendations of the American Psychological Association for the ethical treatment of research participants. The protocol was approved by the Institutional Review Board of the University of Michigan Medical School. All participants gave written informed consent in accordance with the Declaration of Helsinki.

## Author Contributions

SG, JA, IL, and RG contributed to the conception and design of the study. SG oversaw participant recruitment and data collection. JS and RG performed the statistical analysis and preparation of results. SG and JS were the primary writers of the manuscript. All authors contributed to the interpretation of the data, critically revised the manuscript, read and approved the submitted version.

## Conflict of Interest Statement

The authors declare that the research was conducted in the absence of any commercial or financial relationships that could be construed as a potential conflict of interest.
