# Supplementary material for: Diminished Value Discrimination in Obsessive-Compulsive Disorder: A Prospect Theory Model of Decision-Making Under Risk
Source: Front Psychiatry. 2019 Jul 8;10:469. doi: 10.3389/fpsyt.2019.00469 (PMC6628634; doi:10.3389/fpsyt.2019.00469)
Supplement: Supplementary file 1 [file DataSheet_1.pdf]

Supplementary Materials

Multiple sets of starting values were tested to determine the values that provided the best model fit, based on the lowest absolute Log-Likelihood Estimation (LLE) value. Chosen starting values were: α = 1.2, λ = 0.82, γ = 0.672, δ = 1.672. Starting values for all dummy parameters were fixed to zero. Argument values that were used in the nlme function were: maxIter = 450, msMaxIter = 450, niterEM = 450, pnlsMaxIter = 400 (Pinheiro, Bates, DebRoy, Sarkar, & R_Core_Team, 2016). The resulting LLE value of this model was -4862.

References

Pinheiro, J., Bates, D., DebRoy, S., Sarkar, D., & R_Core_Team. (2016). nlme: Linear and Nonlinear Mixed Effects Models. *R package version 3.1-128*, http://CRAN.R-project.org/package=nlme.
